# Molecular alterations in clinical stage III cutaneous melanoma: Correlation with clinicopathological features and patient outcome

**DOI:** 10.3892/ol.2014.2122

**Published:** 2014-05-08

**Authors:** PIOTR RUTKOWSKI, ALEKSANDRA GOS, MONIKA JURKOWSKA, TOMASZ ŚWITAJ, WIRGINIUSZ DZIEWIRSKI, MARCIN ZDZIENICKI, KONRAD PTASZYŃSKI, WANDA MICHEJ, ANDRZEJ TYSAROWSKI, JANUSZ A. SIEDLECKI

**Affiliations:** 1 Department of Soft Tissue/Bone Sarcoma and Melanoma, Maria Sklodowska-Curie Memorial Cancer Center and Institute of Oncology, Warsaw 02-781, Poland; 2 Department of Molecular Biology, Maria Sklodowska-Curie Memorial Cancer Center and Institute of Oncology, Warsaw 02-781, Poland; 3 Department of Biochemistry and Molecular Biology, Institute of Rheumatology, Warsaw 02-637, Poland; 4 Department of Pathology, Maria Sklodowska-Curie Memorial Cancer Center and Institute of Oncology, Warsaw 02-781, Poland; 5 Department of Pathology, Center of Postgraduate Medical Education, Warsaw 01-809, Poland

**Keywords:** melanoma, lymph node, molecular factors, metastases, v-raf murine sarcoma viral oncogene homolog B1, neuroblastoma RAS viral (v-ras) oncogene homolog

## Abstract

The aim of the present study was to evaluate the frequency and type of oncogenic v-raf murine sarcoma viral oncogene homolog B1 (*BRAF)*/neuroblastoma RAS viral (v-ras) oncogene homolog (*NRAS)* mutations in cutaneous melanoma with clinically detected nodal metastases (stage IIIB and C) in relation to clinicopathological features and outcome. The clinicopathological data of 250 patients following therapeutic lymphadenectomy (LND) between 1995 and 2010, as well as *BRAF*/*NRAS* mutational status in corresponding nodal metastases, were analyzed. The median follow-up time was 53 months. *BRAF* mutations were detected in 154 (62%) cases (141 p.V600E, nine p.V600K and four others) and mutually exclusive *NRAS* mutations were detected in 42 (17%) cases. The presence of a *BRAF* mutation was found to correlate with patients of a younger age. The five-year overall survival (OS) rate was 33 and 43% for LND and primary tumor excision, respectively, and the five-year disease-free survival (DFS) rate for LND was 25%. No correlation was identified between *BRAF*/*NRAS* mutational status and RFS or OS (calculated from the date of the LND and primary tumor excision); for *BRAF*- and *NRAS*-mutated melanoma, the prognosis was the same for patients with wild-type (WT) melanoma. The important factors which had a negative impact on OS and DFS were as follows: Male gender, >1 metastatic lymph node and extracapsular extension of nodal metastases. The interval between the diagnosis of the initial melanoma to regional nodal metastasis (median, 10 months) was not significantly different between *BRAF*-mutant and -WT patients. Our largest comprehensive molecular analysis of clinical stage III melanoma revealed that *BRAF* and *NRAS* mutational status is not a prognostic marker in stage III melanoma patients with macroscopic nodal involvement, but may have implications for potential adjuvant therapy.

## Introduction

Metastases to the regional lymph nodes are the most common first clinical manifestation of disease dissemination following the excision of the primary tumor or in unknown primary melanomas (1). Clinically detected nodal metastases are classified as one subgroup (macrometastases) in stage III disease according to the American Joint Committee on Cancer (AJCC) classification version 7.0 (2009) (2,3). However, it is a generally heterogeneous group of patients in terms of prognosis following surgical therapy (2), without the possibility of discrimination between patients with an aggressive and more indolent course of disease based on classical pathological features. The requirement for a fresh characterization of this group of patients is clear to determine novel and reliable prognostic and predictive factors, which may lead to individualized therapeutic approaches.

The past decade has observed significant advances in the understanding of the genetic changes that drive melanoma cells. In addition, there is increasing evidence that melanoma is a genetically complex disease which arises from the accumulation of genetic abnormalities within melanocytes. The constitutive hyperactivation of the RAS/RAF/MEK/ERK pathway has been identified in the majority of melanomas as the critical player in the regulation of cell proliferation, invasion and survival (4–8). This genetic background is commonly achieved via oncogenic mutations in the following two genes: v-raf murine sarcoma viral oncogene homolog B1 (*BRAF)*; or neuroblastoma RAS viral (v-ras) oncogene homolog (*NRAS)*. The occurrence of these activated mutants is mutually exclusive (7), suggesting functional redundancy. The reported frequency of *BRAF* mutations varies between 40 and 70% in cutaneous melanoma (5,6,9) and these are most frequently detected in tumors occurring in skin that is not chronically damaged by the sun (6). To date, >50 distinct mutations in *BRAF* have been identified, however, ~90% of *BRAF* mutants in melanoma are single-base transitions (T>A) at position 1,799, leading to the substitution of glutamic acid for valine at codon 600 of the BRAF protein (p.V600E) (5,10,11), which leads to a 500-fold increase in its kinase activity. The second most common mutation is p.V600K (16–20% of all *BRAF* mutations), followed by p.V600D/p.V600R (12,13). Mutated *BRAF* is important for melanogenesis, however, *BRAF* p.V600E is not sufficient for the malignant transformation of melanocytes (14) and is an early oncogenic event also found at a high frequency in benign nevi (15).

*NRAS* mutations are present in 15–30% of melanomas of the skin (16,17), with codon 61 most commonly altered. Although it has been demonstrated in experimental models that a mutation in *NRAS* is capable of inducing melanoma in Cdkn2a-deficient mice (4), NRAS mutations occur in the congenital nevi at a similar frequency to melanoma (18,19). Mutations of the two oncogenes (*BRAF* and *NRAS*) have a well established and powerful predictive role as validated targets in recently developed molecular targeted therapy for melanoma. BRAF inhibitors, such as vemurafenib and dabrafenib, demonstrate clinical benefit in melanomas harboring the *BRAF* p.V600E mutation and MEK inhibitors act in the presence of *BRAF* and *NRAS* mutations (20,21). However, the prognostic role of mutations in these genes requires further confirmation. One study (12) has indicated that the presence of a *BRAF* mutation markedly correlates with inferior survival in a metastatic setting, however, this finding was not paralleled by differences in disease-free survival (DFS) from the time of the primary melanoma diagnosis. An additional study has also implied that the presence of *NRAS* mutations has a negative influence on survival in stage IV melanoma patients (22). Such results for stage III melanoma are contradictory or lacking, and assessment of the prognosis for patients with regional nodal metastases continues to depend on basic pathological features.

The aim of the current study was to determine the *BRAF* and *NRAS* mutational status of nodal metastases in a large homogeneous group of cutaneous melanoma patients (BRAF inhibitor-naive patients with clinically detected regional nodal metastases), and to correlate those results with the clinical data and patient survival.

## Materials and methods

### Patient characteristics

Patients were considered eligible for the study if they had been diagnosed with clinical stage III cutaneous melanoma (stage IIIB according to the AJCC 2010 classification) (3), available tumor tissue and undergone radical lymphadenectomy (LND) at the Department of Soft Tissue/Bone Sarcoma and Melanoma at the Maria Sklodowska-Curie Memorial Cancer Center and Institute of Oncology (CCIO; Warsaw, Poland) between May 1995 and November 2010, following pathological confirmation of palpable regional nodal metastases without distant metastases. Formalin-fixed, paraffin-embedded (FFPE) tumor samples (of melanoma lymph node metastases exclusively) from the CCIO pathological archives were selected for the study. The clinicopathological stage of the patients was determined by pathological evaluation of the primary lesion and dissected lymph nodes, as well as by physical and routine imaging examination. There was access to the complete clinical data, including the dates of the primary tumor excision, LDN, disease relapse, final follow-up or mortality, for all patients. The patient characteristics are summarized in Table I. Radical LNDs (axillary or inguinal) were performed according to the technique described by Karakousis (23). For the ilioinguinal LND, the superficial and deep levels below the inguinal ligament to the level of the aortic bifurcation combined with obturator LND were routinely excised. The patients were not treated with BRAF or MEK inhibitors. In accordance with the EORTC 18952 trial, 68 patients received interferon-α2b and 58 received radiotherapy as adjuvant treatment following the LND [with no significant influence on overall survival (OS) data as reported previously] (24,25). Of the 324 consecutive patients who underwent LND during the analyzed period of time, 250 cases with sufficient data and pathological material were eligible for the study.

The study was approved by the local bioethics committee of Maria Sklodowska-Curie Memorial Cancer Center and Institute of Oncology (no. 3/2012) according to the Good Clinical Practice Guidelines. Patients provided written informed consent.

The patients had not undergone any other preliminary selection and only patients who met all the aforementioned conditions were enrolled in the study. All patients were followed closely with a median follow-up time of 53 months for survivors (range, 4–186 months). Postoperative follow-up consisted of physical examination and routine imaging investigations (chest X-ray, ultrasound of the abdominal cavity and computed tomography imaging, if metastases were suspected). Routinely, surveillance was recommended every three months for the first two years, every four months in year three, every six months for years four and five, and annually thereafter.

### Mutational testing

For the purpose of the study, all lymph node FFPE samples of each patient were revived by a pathologist in order to select blocks (one per patient) with the highest tumor content and best possible material quality. Insufficient tumor content, massive necrosis, blood spills or calcification, as well as no amplification of DNA, were accounted for as the main excluding criteria. In total, 250 paraffin blocks were selected; 220 samples had a tumor content of >90%, and no samples had a tumor content of <10%. Samples were excised from the whole block surface. The genomic DNA was isolated using the Sherlock AX DNA kit (A&A Biotechnology, Gdynia, Poland) and amplified in the following standard polymerase chain reaction (PCR) conditions performed at a final volume of 37.5 μl, containing 50 ng of genomic DNA, 1 unit of Maxima Hot Start Taq DNA polymerase (Thermo Fisher Scientific, Waltham, MA, USA), 0.2 mM of each dNTP, 0.2 M of each primer, 1.5 mM MgCl_2_ and 1× buffer. The amplification was performed as follows: One cycle at 95°C for 4 min; 35 cycles at 94°C for 30 sec, 58°C for 30 sec and 72°C for 30 sec; and a final extension step at 72°C for 7 min with primers designed in-house for *BRAF* exons 11 and 15, and *NRAS* exons 1 and 2. The products were bidirectly sequenced using the BigDye Terminator Cycle sequencing kit and ABI Prism 3100 Genetic Analyzer (both Applied Biosystems, Carlsbad, CA, USA). In order to identify mutations, the sequences were then compared between *BRAF* (GenBank ref.: NM_004333.4) and *NRAS* (GenBank ref.: NM_002524.4).

### Statistical analysis

All statistical analyses were performed using the R 2.15.1 statistical software (R Core Team, 2012; http://www.R-project.org). Contingency tables were analyzed using the χ^2^ test. The non-parametric Mann-Whitney U test was applied for comparisons of two groups with a non-normal distribution.

For survival analysis, the Kaplan-Meier estimator was used with log-rank tests for bivariate comparisons. OS time for the assessment of the prognostic value of clinical, pathological and molecular parameters was calculated from the date of the primary tumor excision or LND, to the date of the most recent follow-up (censored data) or mortality (as in the melanoma AJCC staging system) (2,26). DFS was calculated from the date of the therapeutic LND to the date of the most recent follow-up or disease recurrence.

The following clinical, pathological and molecular parameters were tested as potential factors affecting patient survival: Gender, age (≤40, >40–60 and >60 years), primary tumor Breslow thickness (≤1.00, 1.01–2.00, 2.01–4.00 and >4.00 mm), presence of ulceration of the primary lesion, primary tumor level of invasion (II/III vs. IV/V), localization of LND (inguinal vs. axillary), number of lymph nodes with metastases (1, 2–3 or ≥4), presence of extracapsular invasion in the involved lymph nodes, *BRAF* status [*BRAF*-mutated vs. wild-type (WT); and p.V600E mutation vs. other *BRAF* mutations and vs. wild-type] and *NRAS* status (*NRAS*-mutated vs. WT). P<0.05 was considered to indicate a statistically significant difference.

## Results

### Mutational status and correlation with clinicopathological features

*BRAF* mutations were detected in 154 of 250 (61.6%) melanoma nodal metastases and were predominantly p.V600E mutations (Table II). The *NRAS* gene was altered in 42 (43.8%) of the *BRAF*-WT samples. Mutations in *BRAF* and *NRAS* were mutually exclusive and 54 samples did not harbor any.

All ‘weak’ sequence peak (<30% of WT signal) mutations were resequenced from the point of PCR reaction and 110 randomly selected samples were reanalyzed from the point of paraffin block dissection, in an independently validated PCR-based in-house diagnostic test, resulting in complete confirmation of the results. Based on our recent study, we estimate that the sensitivity of the approach, concerning V600E mutations, was >98% (27).

Among the clinicopathological features (Table I), the presence of *BRAF* mutations was found to correlate with a younger age of patients (median age, 52 years for *BRAF*-mutated and 60 years for *BRAF*-WT; P<0.01). The opposite correlation was observed for *NRAS*-mutants versus *NRAS*-WT (median age, 61 years for *NRAS*-mutants and 53 years for *NRAS-WT*; P=0.05; data not shown).

### Survival analysis

**Figure 1:**
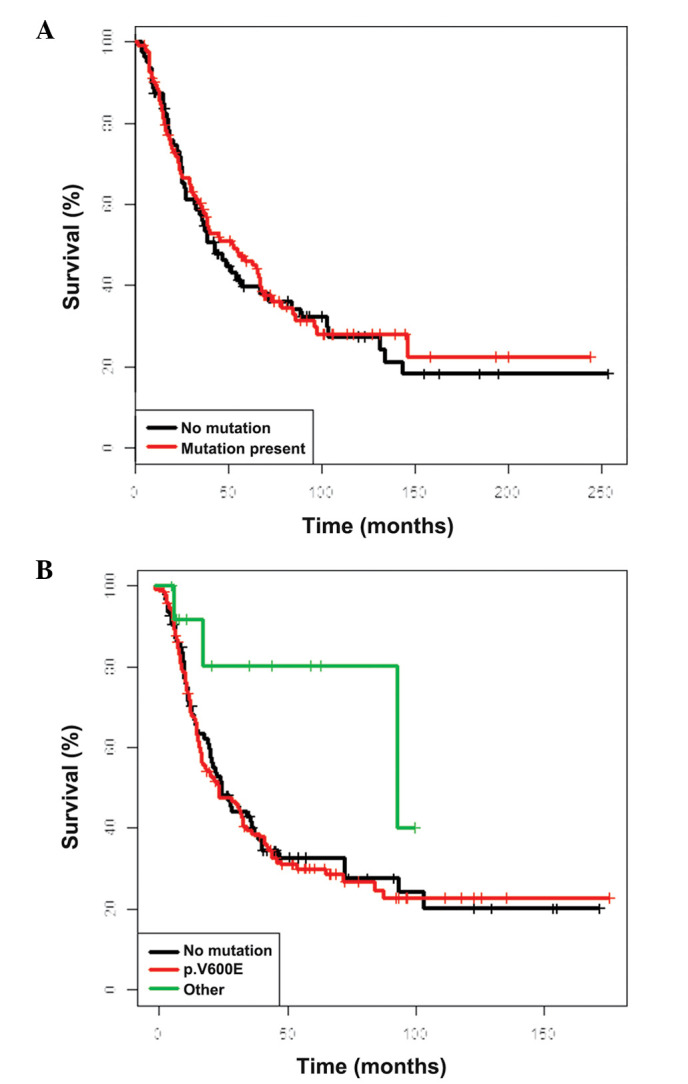
Overall survival according to *BRAF* mutational status in clinical stage III melanoma calculated from (A) the date of the primary tumor excision (*BRAF*-mutants vs. wild-type) and (B) the date of lymph node dissection (*BRAF* p.V600E mutants vs. *BRAF* mutants, with the exception of p.V600E vs. wild-type). *BRAF*, v-raf murine sarcoma viral oncogene homolog B1.

**Figure 2:**
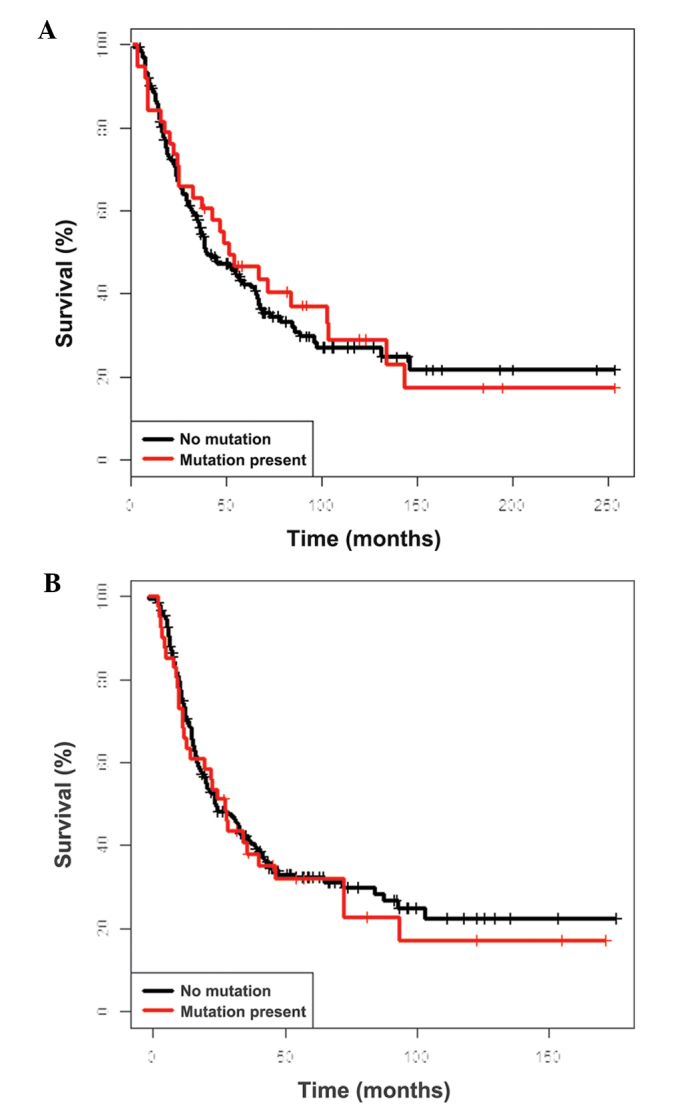
Overall survival according to *NRAS* mutational status in clinical stage III melanoma calculated from (A) the date of the primary tumor excision and (B) the date of lymph node dissection. *NRAS*, neuroblastoma RAS viral (v-ras) oncogene homolog.

Detailed OS data (from the date of the primary tumor excision and LND) are presented in Table IIIA and B. The five-year OS rates for the entire group, calculated from the date of the primary tumor excision and LND, were 43.6 and 32.6%, respectively, and the median survival was 45.5 and 24.3 months, respectively. No correlation was identified between *BRAF* mutational status and OS (calculated from the date of the LND and primary tumor excision) and the prognosis did not differ between *BRAF*-mutated (P=0.73) and *BRAF*-WT (P=0.87) melanomas, however, a trend for an improved OS was identified for non-V600E mutants (Fig. 1). Similarly, *NRAS* mutational status had no impact on survival (Fig. 2). The factors exhibiting a negative impact on OS were: Male gender (P<0.001), >1 metastatic lymph node (P<0.01) and extracapsular extension of nodal metastases (P<0.001).

The interval between the diagnosis of the initial melanoma to regional nodal metastasis was not significantly different between the *BRAF*-mutant and -WT patients (median, 10 months; P=0.29) or between *NRAS*-mutant and -WT patients (median, 10 months; P=0.34).

**Figure 3:**
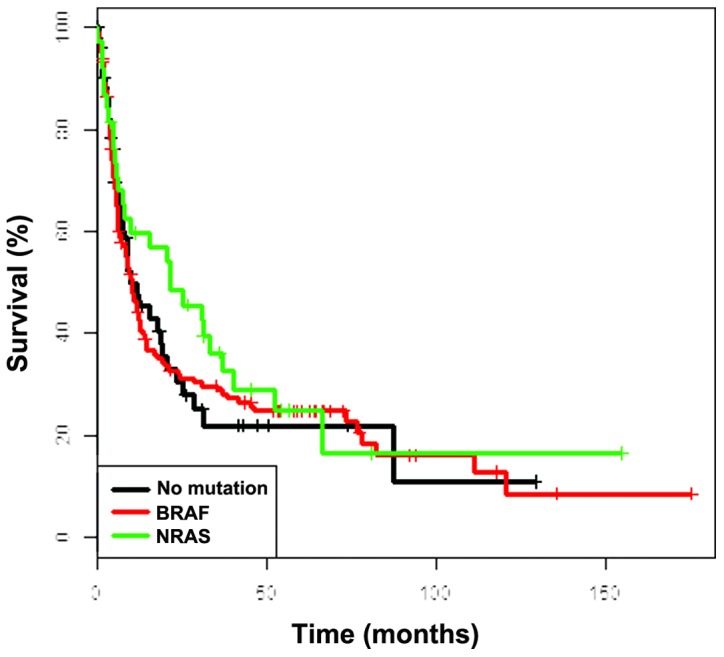
Disease-free survival according to *BRAF* and *NRAS* mutational status calculated from the date of the lymph node dissection. *BRAF*, v-raf murine sarcoma viral oncogene homolog B1; *NRAS*, neuroblastoma RAS viral (v-ras) oncogene homolog.

The five-year DFS rate (from the date of the LND) was 24.6% in the entire group [95% confidence interval (CI), 19.4–31.3] and the median DFS was 12.1 months (95% CI, 9.1–14.8). Similar to the OS, no differences were identified in DFS in relation to *BRAF* and *NRAS* mutational status (Fig. 3), while the clinicopathological factors exhibited similar prognostic significance (data not shown). A total of 181 patients (72%) experienced disease relapse during follow-up. In addition, 135 patients had distant metastases as the first site of recurrent disease; the rates of disease relapse did not differ between the *BRAF*-mutant and -WT patients (75 vs. 68%, respectively; P=0.36) or between the *NRAS*-mutant and -WT patients (71 vs. 73%; P=1.00). In terms of distant metastases, the first relapse site showed a trend towards the presence of brain metastases in *BRAF*-mutated versus -WT patients (19 vs. 9%, respectively).

## Discussion

Employment of individualized therapeutic strategies in the treatment of melanoma requires the identification of reliable prognostic and predictive markers. The present study has expanded the detailed molecular analysis of clinical stage III melanoma by the characterization of *BRAF* and *NRAS* mutations in a homogeneous group of patients with regional nodal macrometastases. The distribution of *BRAF* and *NRAS* mutations in this cohort was similar to that in previously reported studies (particularly consistent with stage IV melanoma) with mutually exclusive *BRAF*-mutants found in 62% and *NRAS*-mutants in 17% of cases (5,12,16,28). These findings confirm that these mutations are early oncogenic events that remain stable throughout disease progression (19,29). Direct sequencing (considered a gold standard in mutation screening) was used as it is the most sensitive method for the detection of rare and/or undefined mutations (30). The tissue material from metastatic nodes was analyzed exclusively, which may be important in the context of future adjuvant treatment.

The results of the current study confirmed former clinical associations with tumor mutational status, but also differed from the observations in stage IV melanoma concerning the role of *BRAF* or *NRAS* mutations as a prognostic marker following complete surgical resection of the metastatic regional lymph nodes. The patients’ age at diagnosis of stage III melanoma was significantly lower in tumors with *BRAF* mutations in contrast to patients with *NRAS* mutations, who were on average older. This is consistent with the observation that *BRAF*-mutated melanomas more frequently affect younger individuals with lower cumulative ultraviolet exposure (16,31,32).

Several studies have reported no influence of *BRAF* or *NRAS* mutations on patient survival from the time of diagnosis (12,16,33,34) in earlier stages of the disease. However, the mutations may impact survival in stage IV disease (12,22,35). The only exception is the study by Moreau *et al* (36), which approached *BRAF* mutational analysis in a heterogenous group of 105 stage III cutaneous melanoma patients and showed a negative prognostic value of *BRAF* mutations. This study had a significantly lower number of cases compared with the cohort of the current study, as well as a shorter follow-up and unusually poor survival (particularly if the authors included the group of patients following positive sentinel node biopsy). The present study showed no difference in patient survival from the primary tumor diagnosis and date of the LND, based on *BRAF* or *NRAS* mutational status. This evidence highlights the role of different genes in melanoma with regional and distant metastases. From the perspective of planned trials with targeted drugs distributed in the adjuvant setting, it is important to note that: i) The genetic abnormalities analyzed in the present study have not altered the natural course of the disease; and ii) the probability of mortality in this group of patients, following conceivably curative surgery, without effective adjuvant therapy may be >60%. The survival in this group of patients is almost identical to that reported in the largest cohort used for validation of the AJCC staging system (3). It appears counter-intuitive that positive *BRAF* status may be associated with a negative prognosis, if the presence of *BRAF* mutations in melanoma closely correlates with a younger age of patients, a well-documented positive prognostic factor in stage I–III melanoma (2,37–39). For non-V600E *BRAF*-mutants, a trend was identified in the current study for a further improved prognosis, which may be associated with a different molecular pathogenesis of this subgroup (40). There remains a requirement for reliable molecular prognostic markers in melanoma (at least in the high-risk stage III), however, in the current study, only established clinicopathological features confirmed their prognostic significance.

In conclusion, the present study represents the largest and most comprehensive molecular evaluation of clinical stage III melanoma undergoing radical LND. The *BRAF* and *NRAS* genotype distribution in the nodal metastases of cutaneous melanomas is identical to that observed in stage IV melanoma, with *BRAF* p.V600E as the most frequent mutation harbored by melanoma cell metastases in lymph nodes. It cannot be confirmed that the *BRAF* and *NRAS* mutations are associated with a more aggressive course of disease, as has been observed in a series of patients with distant metastases (12,22). In the current study, *BRAF* and *NRAS* mutational status was not identified as a prognostic marker in stage III melanoma patients with macroscopic nodal involvement, but had a neutral role in terms of patient survival, which may be of importance for potential adjuvant therapy. *BRAF* and *NRAS* status also had no impact on the disease-free interval from the diagnosis of the primary melanoma to nodal metastases.

## Figures and Tables

**Table I:** Comparison between the patient characteristics of *BRAF*-mutant and -wild-type clinical stage III melanoma.

Patient characteristics	n (%)(n=250)	*BRAF*-mutant, n (%) (n=154)	*BRAF* wild-type, n (%) (n=96)	P-value
Median age, years	54	52	60	P<0.005
Age, years				
0–40	44 (17.6)	26 (16.9)	18 (18.8)	P=0.008
>40–60	118 (47.2)	84 (54.5)	34 (35.4)	
>60	88 (35.2)	44 (28.6)	44 (45.8)	
Gender				
Female	128 (51.2)	76 (49.4)	52 (54.2)	N.S.
Male	122 (48.8)	78 (50.6)	44 (45.8)	
Primary tumor site				
Upper extremity	26 (10.4)	14 (9.1)	12 (12.5)	N.S.
Lower extremity	97 (38.8)	57 (37.0)	40 (41.7)	
Trunk	92 (36.8)	58 (37.7)	34 (35.4)	
Unknown primary	35 (14.0)	25 (16.2)	10 (10.4)	
Lymph nodal basin				
Axillary	122 (48.8)	78 (50.6)	44 (45.8)	N.S.
Inguinal	128 (51.2)	76 (49.4)	52 (55.2)	
Primary melanoma Breslow thickness, mm				
≤1.00	8 (4.2)	6 (5.3)	2 (2.6)	N.S.
1.01–2.00	34 (17.8)	24 (21.2)	10 (12.8)	
2.01–4.00	60 (31.4)	37 (32.7)	23 (29.5)	
>4.00	89 (17.8)	46 (40.8)	43 (55.1)	
Data not available^a^	59	41	18	
Median primary melanoma Breslow thickness, mm	3.9	3.75	4.9	N.S.
Ulceration of primary melanoma				
No	69 (36.3)	38 (33.9)	31 (39.7)	N.S.
Yes	121 (63.7)	74 (66.1)	47 (60.3)	
Data not available^a^	60	42	18	
Metastatic nodes, n				
1	64 (25.6)	40 (25.9)	24 (25.0)	N.S.
2–3	72 (28.8)	46 (29.9)	26 (27.1)	
≥4	114 (45.6)	68 (44.2)	46 (47.9)	
Median	3	3	3	
Extracapsular extension of nodal metastases				
No	114 (45.6)	74 (48.1)	40 (41.7)	N.S.
Yes	136 (54.4)	80 (51.9)	56 (58.3)	

a 35 cases of unknown primary melanoma with nodal metastases.

*BRAF*, v-raf murine sarcoma viral oncogene homolog B1; N.S., not significant.

**Table II:** Oncogenic *BRAF* and *NRAS* mutations within the study group.

Mutation	n (%)	Exon
*BRAF*	154 (61.6)	15
Codon 600	151 (98)	15
p.V600E^a^	141 (91.6)	15
p.V600K	9 (5.8)	15
p.V600D	1 (0.7)	15
Other	3 (2.0)	15
p.E586K	1 (0.7)	15
p.V600_K601delinsE	1 (0.7)	15
p.G469E	1 (0.7)	11
*NRAS*	42 (16.8)	2
Codon 61	40 (95.2)	2
p.Q61R^b^	25 (59.5)	2
p.Q61K	11 (26.2)	2
p.Q61L	3 (7.1)	2
p.Q61H	1 (2.4)	2
Codon 13	2 (4.8)	1
p.G13D	1 (2.4)	1
p.G13R	1 (2.4)	1

Including ^a^139 cases of c.1799T>A single nucleotide transition and two c.1799_1800delTGinsAA complex substitutions, and ^b^two c.181_182delCAinsAG complex substitutions. *BRAF*, v-raf murine sarcoma viral oncogene homolog B1; *NRAS*, neuroblastoma RAS viral (v-ras) oncogene homolog.

**Table III:** Overall survival according to the molecular features of nodal metastases and significant features of primary tumor and nodal metastases (calculated from the date of A, the primary tumor excision and B, the lymph node dissection).

A, Primary tumor excision			

Features	Median survival (95% CI)	Five-year OS rate (95% CI)	P-value
Total group	45.5 (36.9–65.5)	43.6 (37.0–51.4)	
*BRAF* status			
Wild-type	42.7 (32.9–72)	39.8 (29.9–52.9)	0.736
Mutated	53 (36.8–67.9)	46.1 (37.7–56.4)	
*BRAF* codon 600 status			
Wild-type	42.7 (32.9–72)	39.8 (29.9–52.9)	0.098
p.V600E mutants	40.2 (34–66.2)	42.6 (34.1–53.3)	
Non-V600E mutants	96 (96.0–145.1)	90.9 (75.4–100)	
*NRAS* status			
Wild-type	40.2 (36–64.9)	42.5 (35.1–51.4)	0.710
Mutated	51.9 (32.9–134.1)	46.8 (33.2–65.9)	
Ulceration of melanoma			
No	67.4 (38.8–103.8)	55.2 (42.8–71.1)	0.08
Yes	38.6 (29.8–55.6)	36.7 (28.4–47.4)	
Gender			
Female	66.8 (52–97.6)	52.6 (43.8–63.1)	<0.001
Male	29.8 (23.6–44.8)	32.3 (23.3–44.7)	
Age, years			
≤40	72 (42.8–102.3)	57.6 (42.9–77.4)	0.33
<40–60	40.2 (29.8–66.2)	41.1 (32.1–52.6)	
≥60	39.3 (27.6–83.9)	39.7 (28.9–54.6)	
Metastatic lymph nodes, n			
1	64.9 (52.7–105.0)	54.1 (42.0–69.8)	0.01
2–3	54.3 (29.2–104)	46.8 (35.7–61.3)	
≥4	32.6 (24.8–47)	33.0 (23.7–46.1)	
Extracapsular extension of nodal metastases			
No	67.4 (54.3–102.5)	55.3 (45.9–66.6)	<0.0001
Yes	32.6 (24.3–40.2)	30.5 (22.2–41.8)	

B, Lymph node dissection			

Features	Median survival (95% confidence interval)	Five-year OS rate (95% confidence interval)	P-value

Total group	24.3 (20.0–34.2)	32.6 (26.8–39.7)	
*BRAF* status			
Wild-type	24.4 (19.7–38.3)	32.6 (23.7–44.9)	0.867
Mutated	23.5 (16.8–38.8)	32.9 (25.8–42.1)	
*BRAF* codon 600 status			
Wild-type	24.4 (19.7–38.3)	32.6 (23.7–44.9)	0.13
p.V600E mutants	23.4 (16.4–32.7)	30.0 (22.9–39.3)	
Non-V600E mutants	83 (82.0–93.0)	80.2 (58.7–100)	
*NRAS* status			
Wild-type	23.5 (19.7–36.1)	32.3 (26.0–40.3)	0.697
Mutated	27.4 (14.2–72.1)	32.0 (20.1–50.9)	
Ulceration of melanoma			
No	38.8 (27.5–93.0)	37.6 (25.7–55.0)	0.08
Yes	21.7 (16.4–31.2)	27.2 (19.8–37.3)	
Gender			
Female	36.1 (28.1–64.8)	41.0 (32.9–51.1)	<0.001
Male	16.8 (13.9–23.8)	22.6 (15.3–33.4)	
Age, years			
≤40	32.4 (23.0–44.8)	34.2 (21.9–53.4)	0.59
<40–60	23.4 (16.6–38.3)	31.5 (23.8–41.6)	
≥60	20.7 (14.6–40.9)	34.9 (25.1–48.5)	
Metastatic lymph nodes			
1	42 (31.2–75.3)	37.2 (26.0–53.1)	0.005
2–3	23 (14.5–72.1)	40.0 (29.8–53.7)	
≥4	19.2 (14.3–24.5)	24.1 (16.6–34.9)	
Extracapsular extension of nodal metastases			
No	40.9 (31.2–83.8)	41.7 (33.0–52.5)	<0.0001
Yes	17.6 (14.2–23.4)	23.4 (16.4–33.4)	

CI, confidence interval; OS, overall survival. BRAF, v-raf murine sarcoma viral oncogene homolog B1; NRAS, neuroblastoma RAS viral (v-ras) oncogene homolog.

## References

[1] Leiter U, Meier F, Schittek B, Garbe C (2004). The natural course of cutaneous melanoma. J Surg Oncol.

[2] Balch CM, Gershenwald JE, Soong SJ (2010). Multivariate analysis of prognostic factors among 2,313 patients with stage III melanoma: comparison of nodal micrometastases versus macrometastases. J Clin Oncol.

[3] Balch CM, Gershenwald JE, Soong SJ (2009). Final Version of 2009 AJCC Melanoma Staging and Classification. J Clin Oncol.

[4] Hocker TL, Singh MK, Tsao H (2008). Melanoma genetics and therapeutic approaches in the 21st century: moving from the benchside to the bedside. J Invest Dermatol.

[5] Davies H, Bignell GR, Cox C (2002). Mutations of the BRAF gene in human cancer. Nature.

[6] Curtin JA, Fridlyand J, Kageshita T (2005). Distinct sets of genetic alterations in melanoma. N Engl J Med.

[7] Garrido MC, Bastian BC (2010). KIT as a therapeutic target in melanoma. J Invest Dermatol.

[8] Kumar R, Angelini S, Snellman E, Hemminki K (2004). BRAF mutations are common somatic events in melanocytic nevi. J Invest Dermatol.

[9] Gorden A, Osman I, Gai W (2003). Analysis of BRAF and N-RAS mutations in metastatic melanoma tissues. Cancer Res.

[10] Satyamoorthy K, Li G, Gerrero MR (2003). Constitutive mitogen-activated protein kinase activation in melanoma is mediated by both BRAF mutations and autocrine growth factor stimulation. Cancer Res.

[11] Gray-Schopfer VC, da Rocha Dias S, Marais R (2005). The role of B-RAF in melanoma. Cancer Metastasis Rev.

[12] Long GV, Menzies AM, Nagrial AM (2011). Prognostic and clinicopathologic associations of oncogenic BRAF in metastatic melanoma. J Clin Oncol.

[13] Rubinstein JC, Sznol M, Pavlick AC (2010). Incidence of the V600K mutation among melanoma patients with BRAF mutations, and potential therapeutic response to the specific BRAF inhibitor PLX4032. J Transl Med.

[14] Michaloglou C, Vredeveld LC, Soengas MS (2005). BRAFE600-associated senescence-like cell cycle arrest of human naevi. Nature.

[15] Pollock PM, Harper UL, Hansen KS (2003). High frequency of BRAF mutations in nevi. Nat Genet.

[16] Edlundh-Rose E, Egyházi S, Omholt K, Mansson-Brahme E, Platz A, Hansson J, Lundeberg J (2005). NRAS and BRAF mutations in melanoma tumours in relation to clinical characteristics: a study based on mutation screening by pyrosequencing. Melanoma Res.

[17] Goel VK, Lazar AJ, Warneke CL, Redston MS, Haluska FG (2006). Examination of mutations in BRAF, NRAS, and PTEN in primary cutaneous melanoma. J Invest Dermatol.

[18] Poynter JN, Elder JT, Fullen DR (2006). BRAF and NRAS mutations in melanoma and melanocytic nevi. Melanoma Res.

[19] Omholt K, Platz A, Kanter L, Ringborg U, Hansson J (2003). NRAS and BRAF mutations arise early during melanoma pathogenesis and are preserved throughout tumor progression. Clin Cancer Res.

[20] Chapman PB, Hauschild A, Robert C (2011). Improved survival with vemurafenib in melanoma with BRAF V600E mutation. N Engl J Med.

[21] Hauschild A, Grob JJ, Demidov LV (2012). Dabrafenib in BRAF-mutated metastatic melanoma: a multicentre, open-label, phase 3 randomised controlled trial. Lancet.

[22] Jakob JA, Bassett RL, Ng CS (2012). NRAS mutation status is an independent prognostic factor in metastatic melanoma. Cancer.

[23] Karakousis CP (1998). Therapeutic node dissections in malignant melanoma. Semin Surg Oncol.

[24] Eggermont AMM, Suciu S, MacKie R (2005). Post-surgery adjuvant therapy with intermediate doses of interferon alfa 2b versus observation in patients with stage IIb/III melanoma (EORTC 18952): randomized controlled trial. Lancet.

[25] Nowecki ZI, Rutkowski P, Michej W (2008). The survival benefit to patients with positive sentinel node melanoma after completion lymph node dissection may be limited to the subgroup with a primary lesion Breslow thickness greater than 1.0 and less than or equal to 4 mm (pT2-pT3). Ann Surg Oncol.

[26] Berd D, Mastrangelo MJ, Sato T (2005). Calculation of survival of patients with stage III melanoma. J Clin Oncol.

[27] Gos A, Jurkowska M, Siedlecki JA, Michej W, Wiater K, Switaj T, Kosela H, Rutkowski P (2013). Comparison between two widely used laboratory methods in *BRAF* V600 mutation detection rate in FFPE clinical samples of stage III cutaneous melanoma metastases to the lymph nodes.

[28] Lee JH, Choi JW, Kim YS (2011). Frequencies of BRAF and NRAS mutations are different in histological types and sites of origin of cutaneous melanoma: a meta-analysis. Br J Dermatol.

[29] Colombino M, Capone M, Lissia A (2012). BRAF/NRAS Mutation Frequencies Among Primary Tumors and Metastases in Patients With Melanoma. J Clin Oncol.

[30] Pont-Kingdon G, Gedge F, Wooderchak-Donahue W (2012). Design and analytical validation of clinical DNA sequencing assays. Arch Pathol Lab Med.

[31] Liu W, Kelly JW, Trivett M (2007). Distinct clinical and pathological features are associated with the BRAF(T1799A(V600E)) mutation in primary melanoma. J Invest Dermatol.

[32] Viros A, Fridlyand J, Bauer J, Lasithiotakis K, Garbe C, Pinkel D, Bastian BC (2008). Improving melanoma classification by integrating genetic and morphologic features. PLoS Med.

[33] Ellerhorst JA, Greene VR, Ekmekcioglu S (2011). Clinical Correlates of NRAS and BRAF Mutations in Primary Human Melanoma. Clin Cancer Res.

[34] Akslen LA, Angelini S, Straume O, Bachmann IM, Molven A, Hemminki K, Kumar R (2005). BRAF and NRAS mutations are frequent in nodular melanoma but are not associated with tumor cell proliferation or patient survival. J Invest Dermatol.

[35] Houben R, Becker JC, Kappel A (2004). Constitutive activation of the Ras-Raf signaling pathway in metastatic melanoma is associated with poor prognosis. J Carcinog.

[36] Moreau S, Saiag P, Aegerter P (2012). Prognostic Value of BRAFV600 Mutations in Melanoma Patients After Resection of Metastatic Lymph Nodes. Ann Surg Oncol.

[37] Rutkowski P, Nowecki ZI, Zdzienicki M (2010). Cutaneous melanoma with nodal metastases in elderly people. Int J Dermatol.

[38] Kretschmer L, Starz H, Thoms KM (2011). Age as a key factor influencing metastasizing patterns and disease-specific survival after sentinel lymph node biopsy for cutaneous melanoma. Int J Cancer.

[39] Macdonald JB, Dueck AC, Gray RJ, Wasif N, Swanson DL, Sekulic A, Pockaj BA (2011). Malignant Melanoma in the Elderly: Different Regional Disease and Poorer Prognosis. J Cancer.

[40] Menzies AM, Haydu LE, Visintin L (2012). Distinguishing clinicopathologic features of patients with V600E and V600K BRAF-mutant metastatic melanoma. Clin Cancer Res.

